# Occult fish bone ingestion inducing cross-site injury: a rare case report of gastric bleeding and jejunal perforation

**DOI:** 10.3389/fmed.2026.1838469

**Published:** 2026-05-13

**Authors:** Ze-Ming Chen, Guo-Li Cao, Xiao Tang, Ri-Yun Gan, Xi-Qiu Yu

**Affiliations:** Department of Gastroenterology, Shenzhen Luohu People’s Hospital, The Third Affiliated Hospital of Shenzhen University, Shenzhen, China

**Keywords:** cross-site injury, fish bone, gastric bleeding, jejunal perforation, occult fish bone ingestion, peritonitis

## Abstract

**Background:**

Fish bone ingestion is a common clinical problem. However, occult ingestion causing concurrent gastric bleeding and jejunal perforation is extremely rare and easily misdiagnosed.

**Case presentation:**

A 72-year-old male presented with upper abdominal pain and hematemesis with no clear history of foreign body ingestion. Abdominal CT revealed a high-density shadow in the stomach, localized gas–fluid accumulation in the small intestine, and minimal surrounding exudation. Emergency gastroscopy failed to identify the source of bleeding. The patient subsequently developed peritonitis, and emergency laparotomy revealed a migratory fish bone that had penetrated the gastric wall and caused secondary jejunal perforation. The patient recovered uneventfully after removal of the fish bone and partial jejunal resection.

**Discussion:**

The mechanism underlying this cross-site injury is as follows: the fish bone first pierced the gastric wall, causing gastric bleeding; it then penetrated the full thickness of the gastric wall and entered the peritoneal cavity, triggering peritonitis; finally, the migrating fish bone punctured the jejunal wall, leading to jejunal perforation and aggravated intra-abdominal inflammation.

**Conclusion:**

In elderly patients with abdominal pain and hematemesis but no clear history of foreign body ingestion, rapidly elevated inflammatory markers, progressive peritoneal irritation, and negative gastroscopy should raise strong suspicion of occult fish bone–induced cross-site gastrointestinal injury (gastric bleeding and jejunal perforation). Timely laparotomy is critical for definitive diagnosis and improved prognosis in patients with peritonitis secondary to jejunal perforation. This case provides valuable clinical insights for the early diagnosis and management of similar atypical gastrointestinal injuries caused by fish bone ingestion.

## 1 Introduction

Fish bone ingestion is a common and global clinical problem, especially in regions with high fish consumption, including coastal areas in Asia, Europe, and North America (1, 2). Fish bones are among the most common sharp foreign bodies causing gastrointestinal injury, with most injuries occurring in the upper gastrointestinal tract, including the esophagus and stomach (1). The main clinical manifestation is usually persistent abdominal pain, and severe cases may be complicated by perforation, peritonitis, or even life-threatening intra-abdominal infection (3–5). Abdominal CT is the first-line imaging modality for diagnosing fish bone–induced gastrointestinal injury, as it can detect high-density foreign bodies and secondary intestinal wall changes (6). However, gastric contents often obscure these high-density shadows (7), which is a major cause of missed or delayed diagnosis in cases of occult fish bone ingestion. Gastroscopy is the gold standard for diagnosing upper gastrointestinal bleeding (8), but tiny puncture wounds on the gastric wall covered by blood or gastric contents can easily be overlooked. In elderly patients whose clinical and laboratory findings deteriorate despite negative endoscopic results, timely surgical intervention is crucial to avoid delays in treating severe complications such as diffuse peritonitis and septic shock caused by unrecognized jejunal perforation or other intestinal injuries. Herein, we report a rare case of gastric bleeding and jejunal perforation caused by occult fish bone ingestion in a 72-year-old male patient. We analyze the diagnostic challenges and the mechanism of cross-site injury, and summarize key clinical experience to provide a reference for the diagnosis and treatment of similar cases.

## 2 Case presentation

A 72-year-old male was admitted to the hospital due to persistent upper abdominal colic for 8 h and hematemesis for 4 h. He denied a clear history of fish bone or other sharp foreign body ingestion. His medical history was significant for 20 years of primary hypertension, for which he had taken regular oral antihypertensive medications. Before admission, the patient had 10 episodes of hematemesis, with an estimated 50–100 mL of dark red gastric content each time.

**Figure 1:**
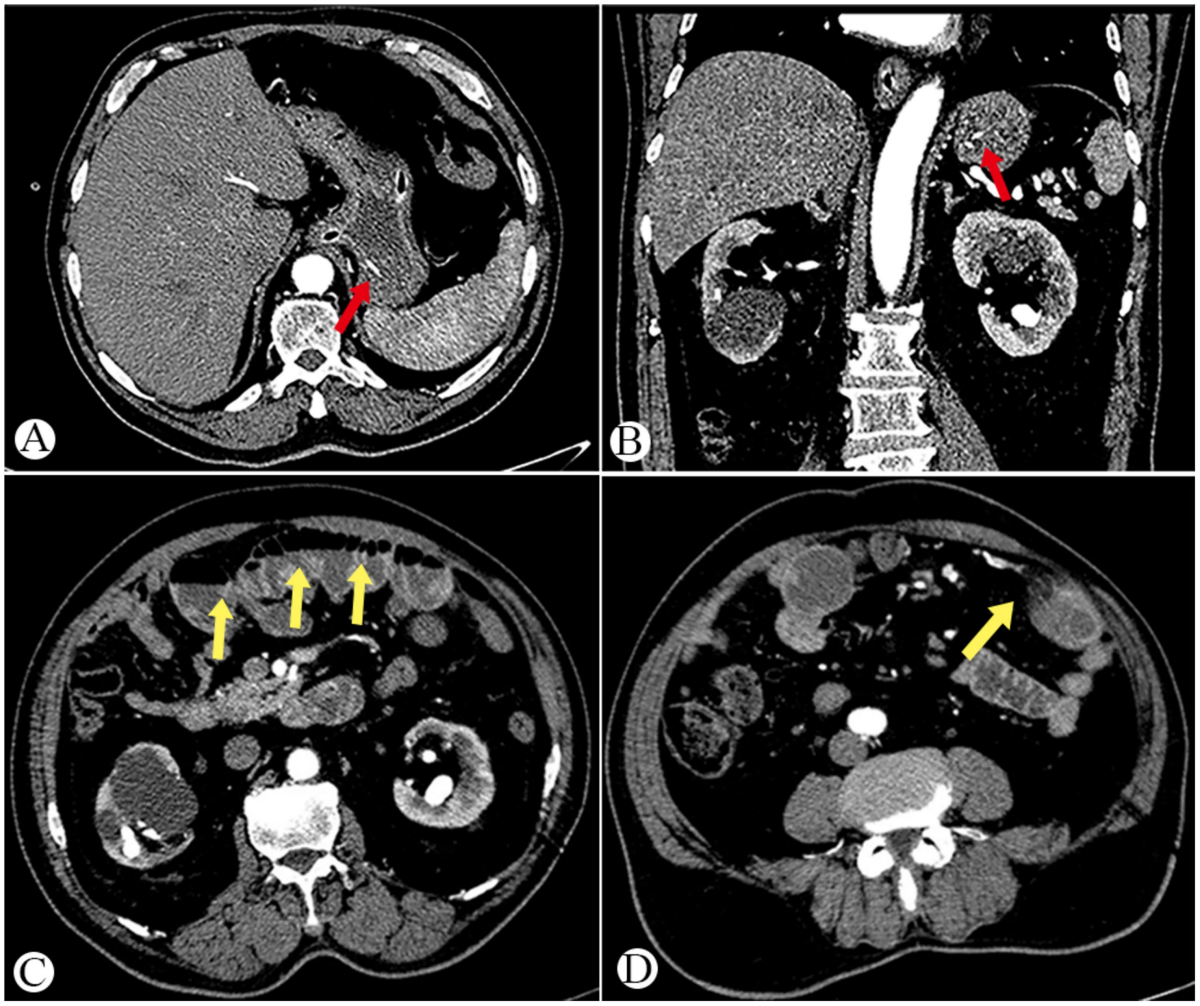
Preoperative abdominal CT findings. **(A,B)** Strip-like high-density foreign body in the gastric cavity (red arrows). **(C)** Local gas-fluid accumulation in the small intestine (yellow arrow). **(D)** Minimal surrounding exudation in the small intestine (yellow arrow).

**Figure 2:**
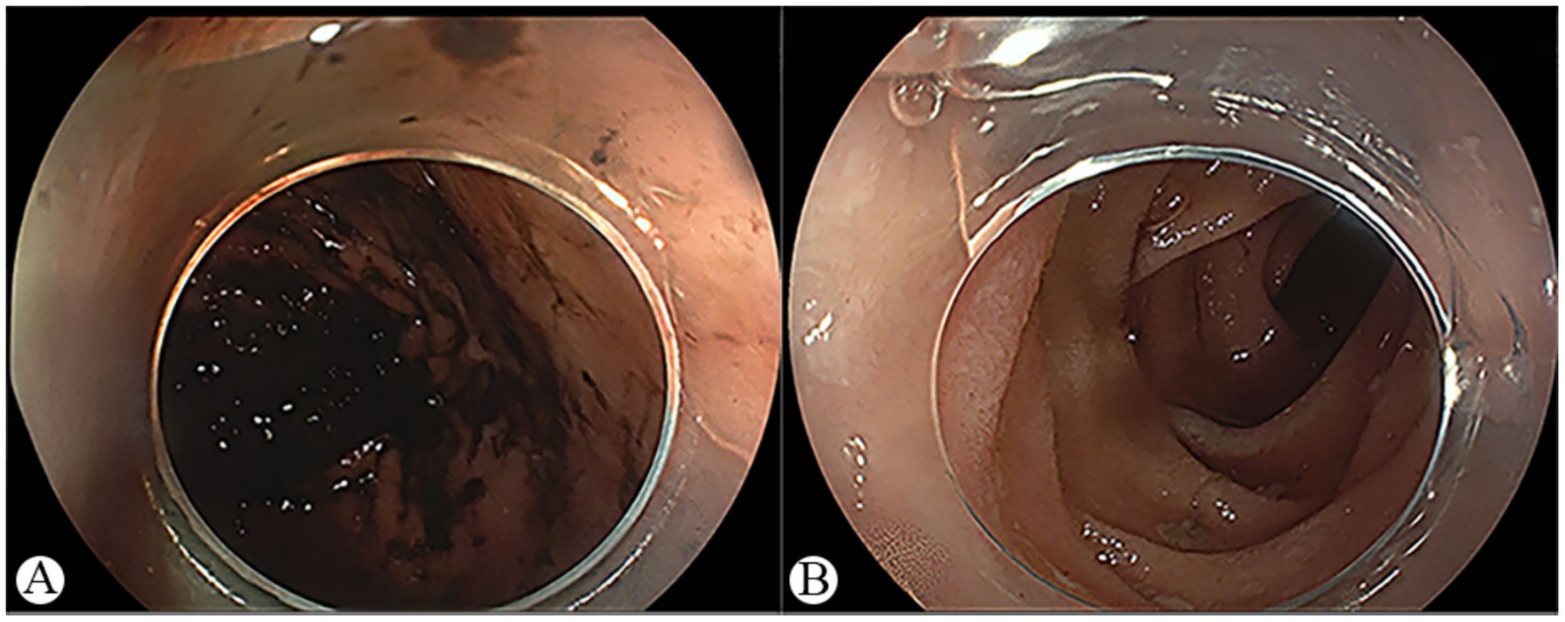
Preoperative gastroscopy findings. **(A)** Large amount of black fluid in the gastric cavity. **(B)** No red fluid in the descending duodenum.

On admission, his vital signs were as follows: body temperature (T) 36.9 °C, blood pressure 159/101 mmHg, heart rate 115 beats per minute, respiratory rate 20 breaths per minute. Physical examination showed signs of anemia, a soft abdomen, and tenderness in the upper abdomen without rebound tenderness. Laboratory tests revealed a white blood cell count of 16.88 × 10^9^/L, hemoglobin 137 g/L, C-reactive protein (CRP) 5.67 mg/L, procalcitonin (PCT) 0.95 ng/mL. The vomitus occult blood test was positive. Abdominal contrast-enhanced CT and angiography showed a large amount of gastric content and strip-like high-density shadows in the gastric cavity (Figures 1A,B), as well as localized gas–fluid accumulation with minimal surrounding exudation in the small intestine (Figures 1C,D). Given the upper gastrointestinal bleeding confirmed by the vomitus occult blood test, emergency gastroscopy was performed 4 h after admission. Gastroscopy showed a large amount of dark fluid in the stomach (Figure 2); after thorough irrigation and careful inspection, no definite active bleeding focus, ulcer, tumor, foreign body, or perforation was found in the esophagus, stomach, or duodenum. After admission, the patient received supportive treatment including proton pump inhibitor therapy, empirical antibiotics, and gastrointestinal decompression.

However, half a day later, the patient developed a low-grade fever (T 37.8 °C) and worsening abdominal pain that progressed from localized upper abdominal pain to diffuse generalized abdominal pain, with no recurrent hematemesis. Vital signs were as follows: T 37.8 °C, blood pressure 147/83 mmHg, heart rate 89 beats per minute, respiratory rate 17 breaths per minute. Physical examination showed significant abdominal distension, muscular rigidity, and diffuse tenderness and rebound tenderness over the entire abdomen. Laboratory tests showed a white blood cell count of 15.94 × 10^9^/L, hemoglobin 107 g/L, CRP 41.22 mg/L, PCT 6.22 ng/mL, indicating progressive intra-abdominal inflammation.

Given the patient’s worsening peritonitis with marked peritoneal irritation, significantly elevated inflammatory markers, and a marked decrease in hemoglobin, combined with the failure of imaging and endoscopy to identify the cause of upper gastrointestinal bleeding and secondary peritonitis, an urgent multidisciplinary consultation was conducted involving gastroenterology, gastrointestinal surgery, and interventional radiology. In view of the clear indication for surgical exploration in unexplained diffuse peritonitis, emergency laparotomy was performed under general anesthesia.

**Figure 3:**
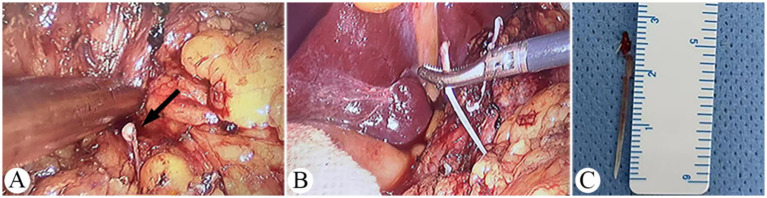
Intraoperative findings. **(A)** Fish bone embedded in the jejunal wall (black arrow). **(B)** Sharp end inside the intestinal lumen and blunt end protruding outward. **(C)** The intact fish bone removed during surgery.

During surgery, approximately 100 mL of purulent exudate was found in the abdominal cavity. Intestinal dilatation was observed approximately 80 cm distal to the proximal end of the jejunum, with mild thickening of the intestinal wall. A fish bone approximately 2.7 cm in length was embedded in the jejunal wall, with its sharp end inside the intestinal lumen and blunt end protruding outward (Figure 3). Purulent exudate was attached to the jejunal perforation site, confirming the diagnosis of jejunal perforation. The patient underwent removal of the fish bone and partial jejunal resection. Postoperatively, he received antibiotics, parenteral nutrition, and other supportive treatments. His condition improved gradually, and he was discharged on postoperative day 15.

## 3 Discussion

This rare case of occult fish bone-induced cross-site gastrointestinal injury is characterized by an unclear ingestion history, atypical early manifestations, multiple diagnostic confounders, and concurrent gastric bleeding, jejunal perforation, and secondary peritonitis. The entire diagnostic and therapeutic process reflects the substantial clinical challenges in managing such rare cases. The mechanism of cross-site injury and the clinical experience gained from this case carry important educational and warning value. We discuss the key points of this case from three perspectives.

### 3.1 Mechanism of cross-site injury induced by occult fish bone ingestion

The complete injury cascade caused by the fish bone was closely associated with gastric bleeding, jejunal perforation, and secondary peritonitis. The elderly patient accidentally ingested the fish bone during eating. Driven by gastric peristalsis, the sharp fish bone pierced the gastric wall, damaging submucosal blood vessels and causing gastric bleeding that manifested as hematemesis. With continued peristalsis and movement of gastric contents, the fish bone fully penetrated the gastric wall and entered the peritoneal cavity. Under the combined effects of local inflammation, fibrous encapsulation, and visceral mobility, the fish bone underwent secondary migration (9, 10). Driven by intestinal peristalsis, the fish bone then punctured the jejunal wall, causing jejunal perforation and leading to peritonitis, which presented as progressive peritoneal irritation and rising inflammatory markers. During gastroscopy, the tiny puncture wound on the gastric wall was obscured by blood and gastric contents, which was the main reason the gastric injury was not detected endoscopically and the cause of bleeding remained unclear in the early stage. Furthermore, the specific anatomical features of the fish bone confirmed at surgery—sharp end inside the lumen and blunt end outside—further clarify the mechanism of jejunal perforation.

### 3.2 Analysis of diagnostic challenges and interference factors

The main diagnostic challenge in this case was the combination of multiple interfering factors, which are also common in the clinical diagnosis of occult fish bone-induced gastrointestinal injury in elderly patients, and represent the key reasons for delayed diagnosis of jejunal perforation and secondary peritonitis. The specific factors are summarized as follows: (1) Occult ingestion with no clear history: Age-related degeneration in chewing, swallowing function (11), and oropharyngeal sensation prevented the patient from recalling fish bone ingestion (12), hindering early linkage of abdominal pain and hematemesis to fish bone injury.g early association of abdominal pain/hematemesis with fish bone injury. (2) Imaging limitations leading to missed diagnosis: Although abdominal CT is valuable, it has limitations in detecting small or deeply embedded fish bones. Small bones may be obscured by intestinal contents, blood, or soft tissue, making clear identification difficult (2). (3) Endoscopic limitations leading to missed diagnosis: Emergency unsedated gastroscopy was limited by the patient’s tolerance and a large amount of dark fluid in the stomach, which prevented complete cleaning and careful inspection of subtle gastric wall lesions, resulting in missed diagnosis of the tiny puncture wound caused by the fish bone. Small or deeply embedded fish bones may be obscured by contents, blood, or soft tissue and may not be visible even on direct endoscopic visualization (2). (4) Atypical initial manifestations masking cross-site injury: Initial abdominal pain and hematemesis mimicked common upper gastrointestinal bleeding (e.g., gastric ulcer), directing clinical attention toward bleeding and away from foreign body-induced mechanical injury and potential cross-site damage.

### 3.3 Clinical insights for diagnosis and treatment

Based on the experience of this case, the key clinical insights for managing occult fish bone–induced cross-site injury are summarized as follows: (1) Strengthen dynamic monitoring: For patients with gastric bleeding and negative gastroscopy, close and continuous monitoring of hemoglobin, inflammatory markers, and abdominal signs is critical for early detection of potential cross-site injury and secondary peritonitis. (2) Timely repeat imaging: For inconclusive initial CT findings, repeat abdominal CT after gastrointestinal decompression; add abdominal plain radiography if perforation is suspected to avoid misdiagnosis from initial imaging. (3) Active surgical exploration: Emergency multidisciplinary consultation and prompt laparotomy are indicated in patients with progressive peritoneal irritation and sharply elevated inflammatory markers of unclear etiology. Laparotomy is both diagnostic and therapeutic and can prevent severe complications such as septic shock (13–15). (4) Heightened vigilance in elderly patients: The elderly are a high-risk group for occult fish bone ingestion (16). Prevention of accidental ingestion and aspiration is particularly important in elderly patients. Caregivers should ensure food is thoroughly deboned, encourage slow eating, and avoid distractions during meals. Enhanced public and clinical education can effectively reduce the incidence of fish bone-related gastrointestinal injury and aspiration events. (5) Treatment decision analysis: For patients with fish bone-induced gastrointestinal perforation and bleeding, conventional treatment options include: a: conservative management for asymptomatic, uncomplicated cases; b: endoscopic removal for intraluminal fish bones without perforation; c: laparoscopic surgery for localized perforation with minimal contamination; d: open laparotomy for unclear diagnosis, generalized peritonitis, or multiple injuries. In this case, emergency open laparotomy was the optimal strategy given the delayed presentation, generalized peritonitis, concurrent gastric bleeding and jejunal perforation, and failure to localize the fish bone preoperatively.

To our knowledge, no previous case has documented sequential gastric bleeding followed by transperitoneal migration and secondary jejunal perforation, making our case extremely rare and clinically instructive. This case report has several limitations. First, as a single case report, the conclusions have limited generalizability and cannot be directly applied to all cases of occult fish bone-induced gastrointestinal injury. Second, the patient had no clear history of fish bone ingestion, so the exact timing and mode of ingestion cannot be determined. Third, the long-term follow-up period is relatively short; thus, the long-term prognosis of the intestinal anastomosis and the risk of late complications such as intestinal adhesion and obstruction cannot be fully evaluated.

## 4 Conclusion

This case report describes a rare case of concurrent gastric bleeding and jejunal perforation as a cross-site gastrointestinal injury caused by occult fish bone ingestion in a 72-year-old male patient. This case provides important clinical insights for the diagnosis and treatment of similar atypical cases. In elderly patients presenting with abdominal pain and gastric bleeding without a clear foreign body ingestion history, highly elevated inflammatory markers, progressive peritoneal irritation, and negative gastroscopy should raise strong suspicion of cross-site gastrointestinal injury caused by occult fish bone ingestion. Dynamic and continuous monitoring, timely re-evaluation of imaging and other auxiliary examinations, and emergency multidisciplinary consultation are key to early diagnosis. Active surgical exploration is an effective approach to confirm the diagnosis, remove the foreign body, repair intestinal perforation, and improve the prognosis of patients with peritonitis secondary to jejunal perforation. This case enriches the clinical spectrum of occult fish bone-induced gastrointestinal injury and provides a valuable reference for the early identification, timely diagnosis, and rational management of similar cross-site injury cases.

## Data Availability

The original contributions presented in the study are included in the article/supplementary material, further inquiries can be directed to the corresponding author.
